# A Passive Temperature-Sensing Antenna Based on a Bimetal Strip Coil

**DOI:** 10.3390/s17040665

**Published:** 2017-03-23

**Authors:** Xianwei Shi, Fan Yang, Shenheng Xu, Maokun Li

**Affiliations:** State Key Laboratory on Microwave and Digital Communications, Tsinghua National Laboratory for Information Science and Technology, Department of Electronic Engineering, Tsinghua University, Beijing 100084, China; shixw14@mails.tinghua.edu.cn (X.S.); shxu@tsinghua.edu.cn (S.X.); maokunli@tsinghua.edu.cn (M.L.)

**Keywords:** reconfigurable sensing antenna, temperature sensing, high sensitivity, bimetal strip

## Abstract

A passive temperature-sensing antenna is presented in this paper, which consists of a meandering dipole, a bimetal strip and a back cavity. The meandering dipole is divided into two parts: the lower feeding part and the upper radiating part, which maintain electric contact during operation. As a sensing component, a bimetal strip coil offers a twisting force to rotate the lower feeding part of the antenna when the temperature varies. As a result, the effective length of the dipole antenna changes, leading to a shift of the resonant frequency. Furthermore, a metal back cavity is added to increase the antenna’s quality factor Q, which results in a high-sensitivity design. An antenna prototype is designed, fabricated, and measured, which achieves a sensitivity larger than 4.00 MHz/°C in a temperature range from 30 °C to 50 °C and a read range longer than 4 m. Good agreement between the simulation and measurement results is obtained.

## 1. Introduction

With the rapid development of Internet of Things (IoT), the concept of reconfigurable sensing antenna (RSA) has emerged in the last decade [1]. The RSAs are the devices that not only can receive and transmit electromagnetic wave just as general antennas do, but also can measure the surrounding environmental parameters like temperature, humidity and gas concentration. Moreover, different from many traditional sensors, the most significant feature of RSAs is passive, or no local power supply, which makes the large-scale deployment of the sensors much more energy-saving. In addition, integrated with Radio Frequency Identification (RFID) technology, the RSAs have the capability of wireless identification. RSAs can be used in many fields such as environmental monitoring, medical treatment, public health, animal husbandry, metal detection and logistics monitoring.

Generally, there are two types of RSAs, with chip and without chip (chipless). In [2], various smart materials for chipless Radio Frequency (RF) sensing applications and their characteristics in the influence of various physical parameters have been analyzed. The operating principle of chipless RFID sensors along with various designs, experimental results, and potential applications is presented. In [3], a chipless sensor for water level detection is introduced.

Meanwhile, the RSA integrated with RFID chip attracts more interest since the sensing information is connected with a 64-bit ID number. In [4,5], moisture RFID sensors are designed with carbon nanotube and sand/soil as the sensing components, respectively, meanwhile Received Signal Strength Indicator (RSSI) and Radar Cross-Section (RCS) are used as the measuring parameters accordingly. Papers [6,7,8] present designs of passive strain sensors, which take the read range and received power as the measuring variables to collect the information of strain force. Besides, there are passive sensors for displacement sensing [9], metal detection [10], capacitive sensing [11], discharge detection [12], tilt sensing [13] and so on. It is worth mentioning that when designing RSAs, the frequency reconfigurable antennas are preferred because compared to RSSI, read range, received power and RCS, the resonant frequency is independent of the location and the orientation of the transponder. In other words, the resonant frequency is more reliable than the other parameters.

In recent years, the frequency reconfigurable temperature-sensing antenna has gained growing attraction. In [14], authors present reconfigurable antennas integrated with thermal switches for wireless temperature monitoring. When the T-matched dipole is integrated with one thermal switch, the sensor can monitor whether the temperature is higher or lower than 40 °C; while integrated with two thermal switches, the sensor can monitor three statuses, i.e., below 40 °C, between 40 °C and 50 °C or above 50 °C. However, the bandwidth of the dipole is relatively broad, which limits the accuracy and results in difficulty for the reader to distinguish two adjacent temperature statuses. Another shortcoming is that the gain of the dipole is small and so the read range of the sensor cannot be far enough. To narrow the bandwidth and improve the gain, papers [15,16] present the design and analysis of the patch-type reconfigurable sensing antennas with one and two thermal switches respectively for temperature monitoring. In particular, a dual-frequency antenna with two thermal switches [16] offers the capability of self-reference.

The aforementioned sensors are for monitoring discrete or threshold temperature. In some scenarios, the continuous temperature sensors are needed. In the design of [17], the temperature sensor can operate in temperature ranges between 20 °C and 370 °C with an average sensitivity of 307 KHz/°C. In [18], authors present a dual-port temperature sensor tag for passive Ultra High Frequency (UHF) RFID systems and give the detailed relationship between the frequency difference and the ambient temperature from 20 °C to 60 °C. The thesis [19] gives detailed introduction of the design and analysis of reconfigurable sensing antennas for wireless sensing applications and makes some concluding suggestions that to obtain a high sensitivity, the sensing antennas should have high gain, large frequency shift and narrow bandwidth. In [20], a sensor which can monitor temperature and pH is presented, but the read range of the High Frequency (HF) sensor is only 4 cm.

In order to improve the sensitivity of passive temperature-sensing antennas, this paper proposes the RSA based on a bimetal strip coil. A rotatable dipole integrated with a RFID chip, a back cavity and a thermal bimetal strip coil are the main parts of the sensor. The initial prototype achieves the highest sensitivity more than 4.00 MHz/°C and a read range more than 4 m.

## 2. Design Methods

### 2.1. A Bimetal Strip Coil for Temperature Sensing

**Figure 1:**
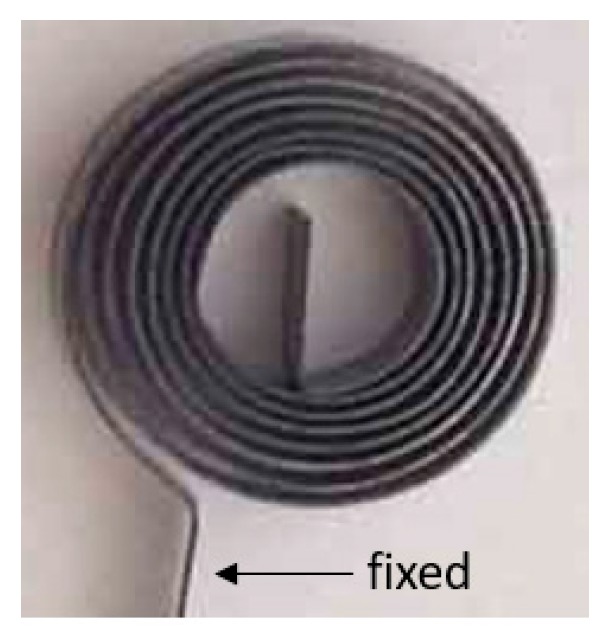
A prototype of a bimetal strip coil.

A bimetal strip is made of two different alloying strips attaching together. Since different alloys have different thermal expansion coefficients, when the temperature varies, the bimetal strip bends. In this work, a P675R bimetal strip which consists of Mn72Cu18Ni10 on one side and Ni36Fe on the other side was used. After processed into a coil and fixed on one end as Figure 1 shows, the bimetal strip coil rotates when the temperature changes. In this design, the bimetal strip coil can achieve a rotation of 100° when the temperature changes from 20 °C to 50 °C without any friction, which is provided by the manufacturer. The bimetal coil will be integrated with an antenna for wireless sensing.

### 2.2. A Dipole Antenna for Wireless Identification and Sensing

**Figure 2:**
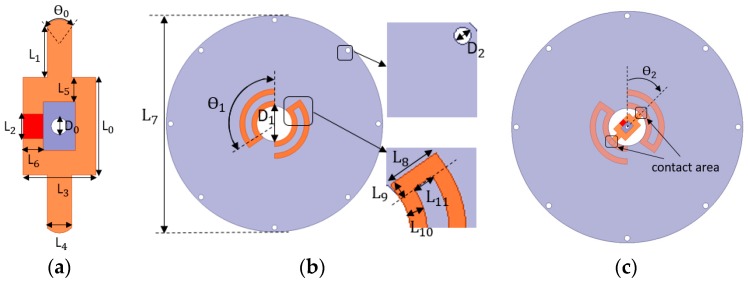
Configuration of the dipole antenna: (**a**) Geometry of the lower feeding part; (**b**) Geometry of the upper radiating part; (**c**) Assembling configuration of the dipole consisting of two parts. The parameter θ_2_ is the rotating angle of the lower part relative to the initial position.

A dipole antenna includes two parts, the lower feeding part (Figure 2a) and the upper radiating part (Figure 2b). Both parts are designed on a RF-60A-0500 substrate with a dielectric constant (ε_r_) of 6.15, a loss tangent of 0.0038, and a thickness of 1.27 mm. The lower part is a feeding loop with a RFID chip. The upper part consists of two curving U-shape arms which are symmetrical relative to the center point of the circular substrate. The curving U-shape arms could reduce the size of the dipole and guarantee the electrical connection between the lower feeding part and the upper radiating part when relative rotation occurs between these two parts. Figure 2c depicts how these two parts work together. When assembling, the center of the lower part aligns with that of the upper part. In Figure 2 the red is a Higgs-3 chip from Alien Technology, the orange is the metal layer, the grey is the substrate, and the tiny circular holes with diameters of D_0_ or D_2_ are used for fixation. The detailed dimensions of the dipole antenna are shown in Table 1.

### 2.3. A Back Cavity for the Antenna Assembly

**Figure 3:**
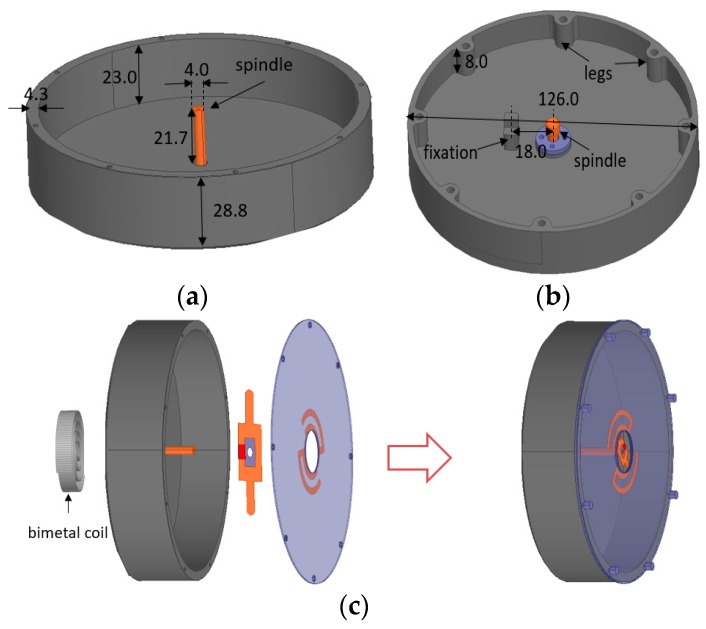
Geometry of the back cavity. (**a**) The 3-D view of the cavity; (**b**) The back view of the cavity; (**c**) The assembly schematic diagram of the proposed antenna. All dimensions are in millimeters.

In order to load a bimetal strip coil into the dipole antenna, an aluminum back cavity is added between them as a supporting structure. The image theory [21] tells that if a horizontal dipole is located too close to a Perfect Electric Conductor (PEC) plane, the radiation will be cancelled by the opposite image of the dipole, so the distance between the bottom of the metal cavity and the dipole should not be too short. In this design, the distance is chosen to be 23 mm. Figure 3a,b gives the geometry of the back cavity and some important dimensions (in millimeters). The spindle can rotate relative to the cavity, the legs with screw are connected to the back test structure, and the fixation is used to fix the bimetal coil.

Figure 3c describes the assembly procedure of the bimetal strip coil, the back cavity and the dipole antenna. One end of the bimetal strip coil is soldered to the fixation and the other is fixed to the spindle. The lower feeding part of the dipole is connected to the other end of the spindle with a screw, and the upper radiating part is fixed to the side wall of the cavity with screws. After assembly, the bimetal strip coil can rotate the lower part of the dipole with the support of the spindle when temperature varies.

## 3. Antenna Simulation Results

### 3.1. Radiation Performance of the Antenna

**Figure 4:**
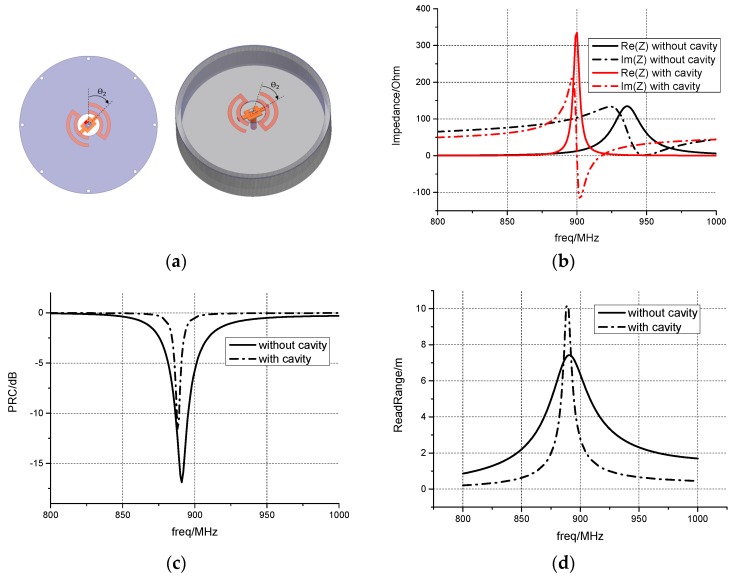
(**a**) The dipole antenna without cavity (left) and the dipole antenna with cavity (right); (**b**) The impedance of the antenna without and with cavity when θ_2_ = 40°; (**c**) The power refection coefficient of antenna without and with cavity when θ_2_ = 40°; (**d**) The read range of the antenna without and with cavity when θ_2_ = 40°.

The antenna is simulated using ANSYS HFSS which is a commercial finite element method (FEM) solver for electromagnetic structure from ANSYS, Inc. (Canonsburg, PA, USA). Without loss of generality, the rotation angle is set to be 40°, and the other rotation angles will be discussed in following sections. The performance comparison between the dipole antenna alone and the dipole antenna with back cavity is given in Figure 4, the power reflection coefficient is defined as:

(1) $$\mathrm{PRC}=20*\log_{10}(\frac{Z_{\mathrm{chip}}{-Z}_{\mathrm{ant}}^{*}}{Z_{\mathrm{chip}}{+Z}_{\mathrm{ant}}}),$$

Z_chip_ is the input impedance of the Higgs-3 chip and Z_ant_ is the input impedance of the antenna. Figure 4b shows the impedance characteristics of the two antennas. The read range D_RSA_ can be calculated according to the definition in [19]:

(2) $$D_{\mathrm{RSA}}=\frac{\lambda }{4\pi }\sqrt{\frac{\mathrm{EIRP}}{P_{\mathrm{IC}}{/G}_{\mathrm{RSA}}}},$$

λ is the operating wavelength. EIRP is the equivalent isotropic radiated power and its value is 3.28 W according to the regulation of European radio standards (EN radio standards). P_IC_, which is the minimum RF communication power of the Higgs-3 chip, equals to −14 dBm according to [22]. G_RSA_ is the realized gain of the RSA antenna.

From Figure 4c, it can be seen that at 886 MHz, PRCs of the two antennas are below −10 dB, which demonstrates the effectiveness of the feeding structure. The 3 dB bandwidth of the antenna with cavity is about 8 MHz, which is obviously narrower than 29 MHz of the antenna without cavity. So, with the cavity, the antenna could obtain a higher quality factor Q. The same results can be found in Figure 4d. Figure 4d also shows that the read range of the antenna with cavity is over 10 m, while that of the antenna without cavity is only about 7.4 m, which is because the cavity enhances the unidirectional radiation due to the existence of the metal plane behind the dipole.

### 3.2. Frequency Shift of the Antenna with the Rotation of the Bimetal Coil

When the temperature changes, the bimetal coil rotates. Meanwhile, the lower feeding part of the antenna rotates because of the twisting force the bimetal coil offers. Therefore, the resonant frequency of the antenna shifts. The sensitivity of the RSA S_T_ can be obtained from:

(3) $$S_{T}{=S}_{A}*S_{C}=\frac{\Delta f}{\Delta \theta }*\frac{\Delta \theta }{\Delta T}=\frac{\Delta f}{\Delta T},$$

**Figure 5:**
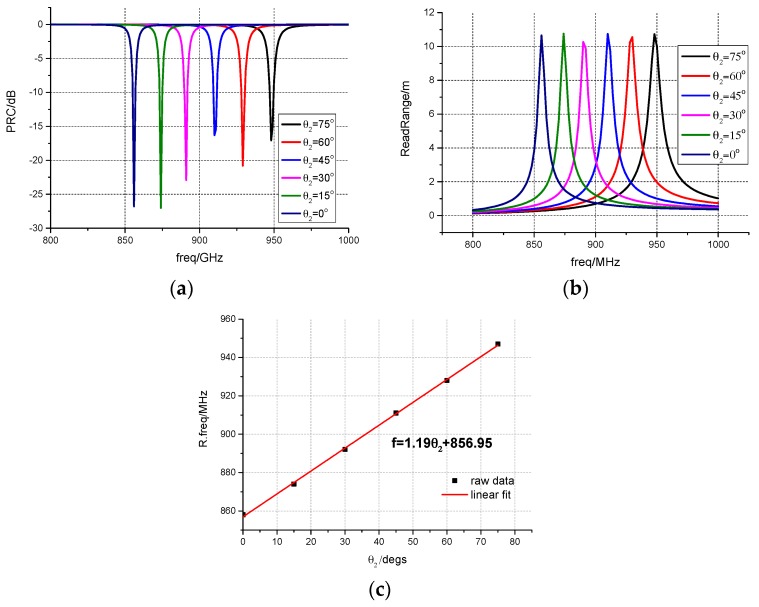
(**a**) The power reflection coefficient at different rotating angles; (**b**) Read range at different rotating angles; (**c**) The relation between the resonant frequency and the rotating angle θ_2_.

The unit is MHz/°C. S_A_ is the rate of change of the antenna’s resonant frequency with the rotation angle, and S_C_ is the change rate of the bimetal coil’s rotation angle with temperature. In this work, the S_C_ of the bimetal coils is 3.33°/°C. Figure 5a,b depicts the simulated results about the frequency-shifting characteristics of the antenna. The read range of the proposed antenna reaches 10 m for all rotation angles. When θ_2_ varies from 0° to 75°, the resonant frequency shifts from 856 MHz to 950 MHz. S_A_ is calculated to be 1.19 MHz/°, and a nearly linear relationship between the rotating angle θ_2_ and the resonant frequency is achieved (Figure 5c). The theoretical sensitivity S_T_ of the proposed antenna is about 3.97 MHz/°C according to the Equation (3). The red line in Figure 5c is the linear fitting line of the raw data.

## 4. Antenna Prototypes and Measurement

### 4.1. Prototypes of the Temperature-Sensing Antenna

**Figure 6:**
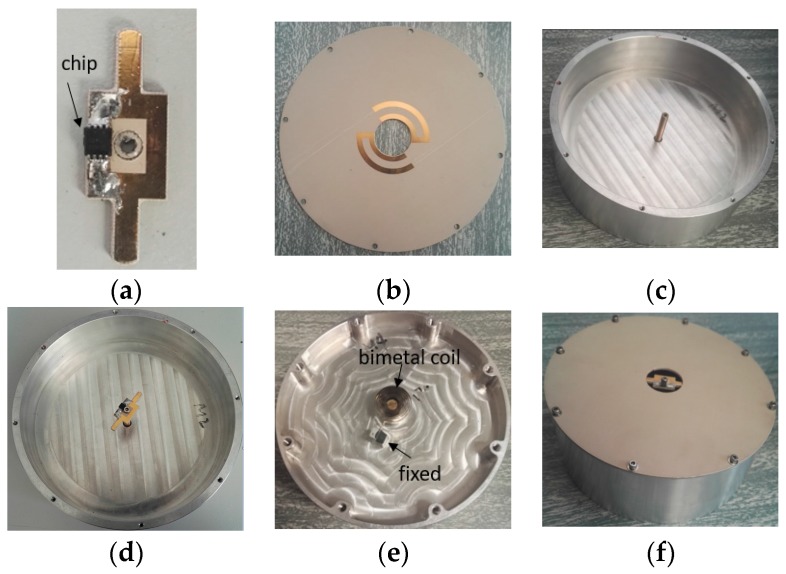
(**a**) The lower feeding part; (**b**) The upper radiating part; (**c**) The cavity; (**d**) Top view and (**e**) bottom view of the cavity integrated with the lower feeding part and the bimetal coil; (**f**) 3-D view of the proposed antenna.

Prototypes of all components of the proposed antenna, including the lower feeding part, the upper radiating part, and the cavity, are shown in Figure 6a–c respectively. The three parts and the bimetal strip are assembled together as Figure 3c shows. Figure 6d,e show the top view and the bottom view of the cavity integrated with the lower feeding part and the bimetal coil. Figure 6f shows the prototypes of the proposed antenna in 3-D view.

### 4.2. Measurement of the Proposed Antenna

**Figure 7:**
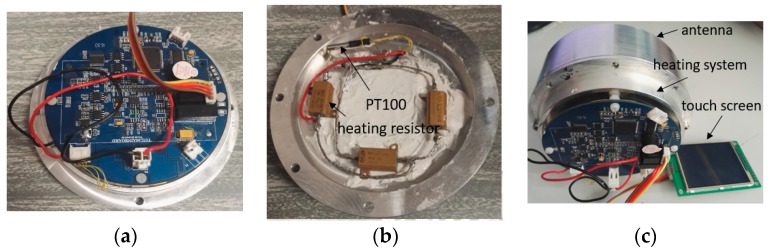
(**a**) Bottom view of the heating system; (**b**) Top view of the heating system; (**c**) Back view of the proposed antenna integrated with the heating system.

In order to measure the performance of the proposed antenna, a heating system is designed and fabricated. This heating system can achieve a heating range from 20 °C to 70 °C and a temperature resolution of 2 °C. The prototype of the heating system is shown in Figure 7. The temperature-controlling circuit is shown in Figure 7a. In Figure 7b, three resistors are used for heating and a PT100 temperature sensor is used for monitoring the temperature inside the cavity. The heating system connects to a touch screen so that the processes of heating and cooling could be displayed. In Figure 7c, the heating system is connected to the back of the cavity to provide a controllable temperature environment for testing the sensing antenna.

**Figure 8:**
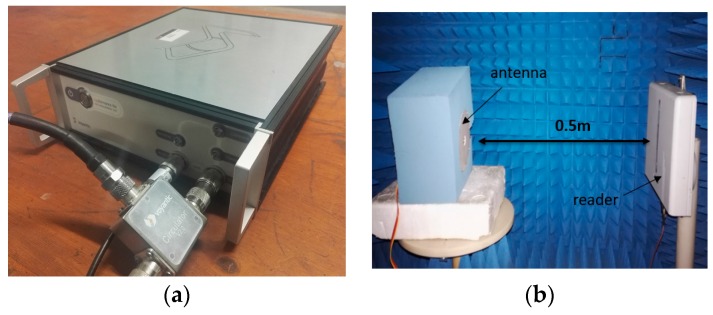
(**a**) The Voyantic Tagformance lite UHF RFID measurement system; (**b**) Measurement setup in an anechoic chamber.

The reader used for the measurement of the proposed sensing antenna has to meet the following two functions: sweeping frequency with a certain range of transmitted power and obtaining the receiving signal strength. A Voyantic Tagformance lite UHF RFID measurement system, which has the above two functions, is used to measure the radiation performance of the proposed antenna. Figure 8a shows the photo of the Voyantic Tagformance lite. In an anechoic chamber (Figure 8b), the distance between the antenna under test and the reader is 0.5 m. Inside the reader there is an antenna with linear polarization for transmitting electromagnetic wave to, and receiving electromagnetic wave from, the sensing antenna. The heating process is controlled by the heating system, which is connected to a power source outside the chamber by an electric wire. In the test, the heating system is used to provide a specified temperature inside the antenna cavity. The UHF RFID measurement system is used to obtain the read range of the antenna [22].

**Figure 9:**
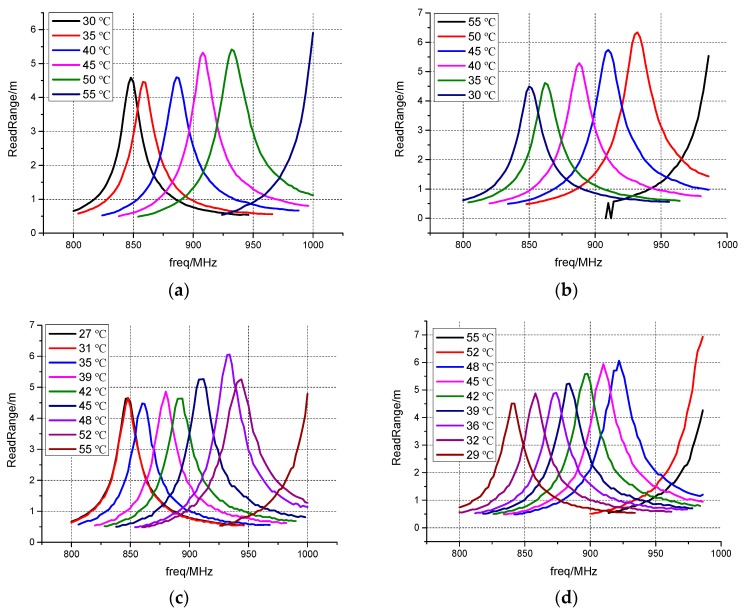
Measured read ranges in (**a**) heating process with temperature step 5 °C; (**b**) cooling process with temperature step 5 °C; (**c**) heating process with temperature step 3 °C; (**d**) cooling process with temperature step 3 °C.

The measurement process and the results are shown in Figure 9. First, the temperature step is set to be 5 °C and the temperature varies from 30 °C to 55 °C. In the heating process (Figure 9a) and the cooling process (Figure 9b), the measurement is carried on respectively. Next, the temperature step is set to be 3 °C and the measurement is repeated in the heating (Figure 9c) and cooling (Figure 9d) processes.

It can be seen that when the temperature step is 5 °C, an average of 23 MHz frequency shift between two adjacent resonant frequencies is obtained in both the heating process and the cooling process. When the temperature step is 3 °C, the average frequency shift between two adjacent resonant frequencies decreases to 13 MHz, but it is still enough to distinguish the adjacent resonant frequencies. In both temperature steps, the read range is more than 4.5 m.

However, when the temperature step is 3 °C, in the heating process, 27 °C curve overlaps 31 °C curve, and in the cooling process, 55 °C curve overlaps 52 °C curve. The reason is that when the temperature varies 3 °C, the twisting force resulting from the deformation of the bimetal coil is not enough to prevail over the starting friction. However, once the friction is overcome in the same process, the frequency shift is sufficient to make the resonant frequencies distinguishable.

**Figure 10:**
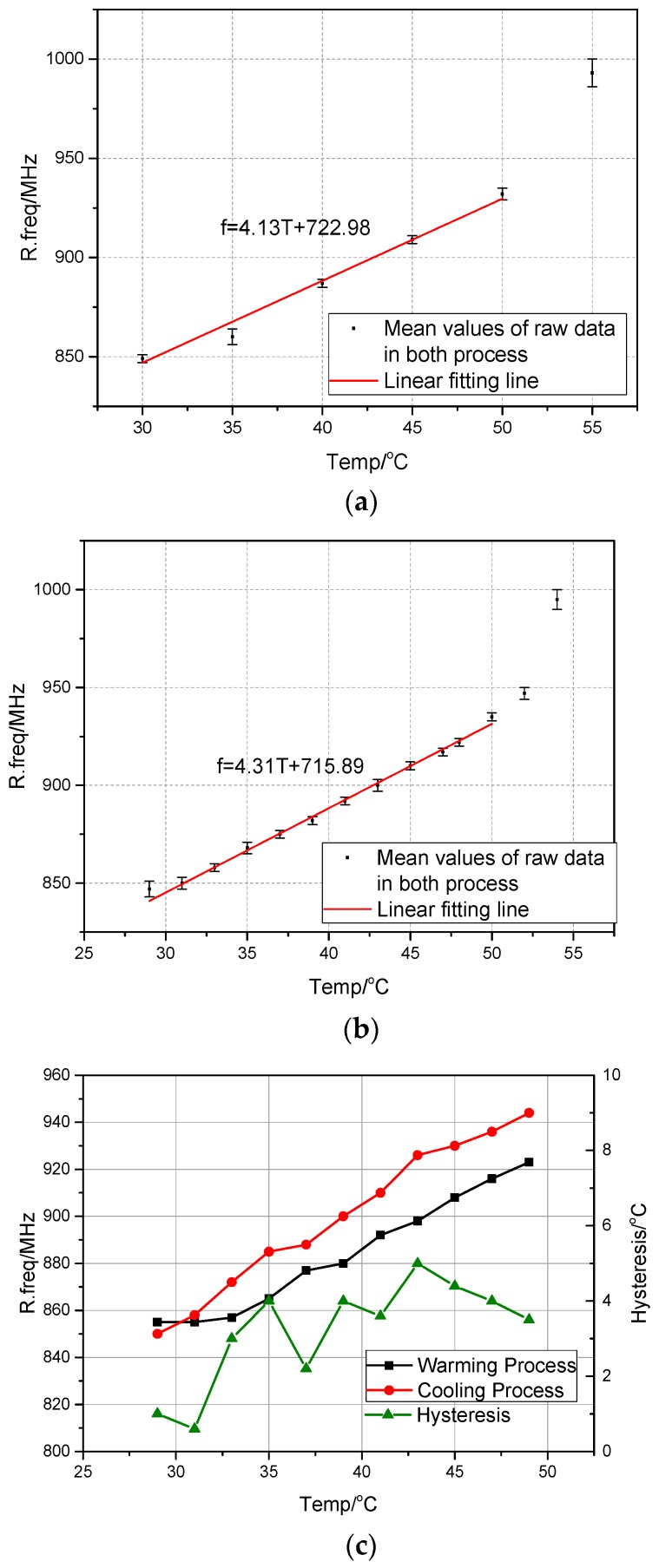
The frequency shift curves and the linear fitting curves when (**a**) the temperature step is 5 °C; (**b**) the temperature step is 2 °C; (**c**) Hysteresis characteristics of the sensing antenna.

Figure 10a,c shows the mean values of the measured resonant frequency at different temperatures. When the temperature step is 5 °C, the average sensitivity S_T5_ is about 4.13 MHz/°C and when the temperature step is 2 °C, the average sensitivity S_T2_ equals to 4.31 MHz/°C. S_T5_ and S_T2_ are close to the theoretical sensitivity S_T_ 3.97 MHz/°C. It is noted that when the temperature is over 50 °C, which is beyond the operation temperature range of the bimetal coil, the measured resonant frequencies increase at a faster rate. Figure 10a,b also shows the linear fitting curves of the measured data. It can be seen that the temperature and the resonant frequency achieve appropriately linear relation. In Figure 10c, the hysteresis behavior of the proposed sensing antenna in heating and cooling processes are presented. The hysteresis *H_t_* at temperature *t* is calculated by:

(4) $$H_{t}=\frac{\left|f_{ct}-f_{ht}\right|}{S_{T2}},$$

where *f_ct_* and *f_ht_* are the resonant frequencies of the sensing antenna in cooling and heating processes, respectively, when the temperature is *t*. The hysteresis of the sensing antenna is evaluated in the anechoic chamber, where the temperature is increased from 29 °C to 55 °C, and then decreased to 29 °C. The sensing antenna exhibits an average hysteresis of 3 °C with a maximum hysteresis of 5 °C at 43 °C. The hysteresis mainly results from the friction between the lower matching part and the upper radiating part. This can be improved by using a bimetal coil with a stronger torque or designing a non-contact coupling structure between the matching part and the radiating part.

### 4.3. Comparison between Simulation Results and Measurement Results

**Figure 11:**
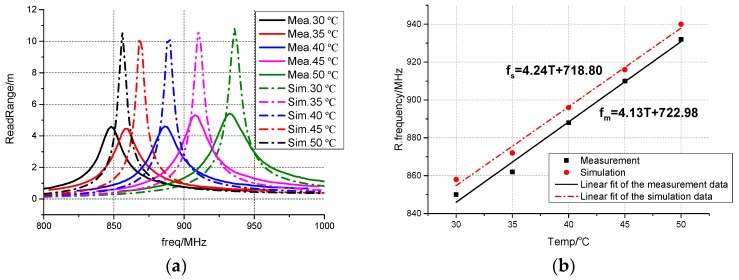

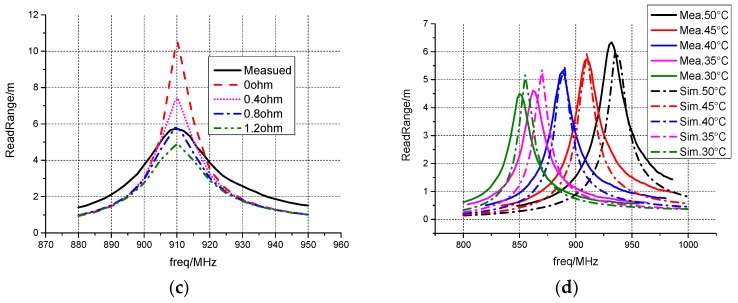
(**a**) Simulated and measured read range; (**b**) Simulated resonant frequency and measured resonant frequency at different temperatures; (**c**) Read range at 45 °C when the cascaded resistor varies; (**d**) Simulated and measured read range with cascaded 0.8 ohm resistors.

Using a standard protractor with accuracy of 1°, the rotating angles at different temperatures can be measured. The relationship between them is shown in Table 2. With the correspondence, the simulated results at the corresponding temperature are obtained. The comparison between the simulated results and the measured results when the temperature step is 5 °C is shown in Figure 11. It can be seen from Figure 11a,b that in both the simulation and the measurement results, when the temperature rises, the resonant frequency of the sensing antenna shifts upward and vice versa. Moreover, the temperature and the resonant frequency show an approximately linear relationship with the temperature from 30 °C to 50 °C in the simulation results and the measurement results. 

There are two major discrepancies between the simulation and the measurement results, namely, the resonant frequency shift and the difference of read range. The measurement resonant frequency slightly shifts downward (0.9%) compared with the simulation results, which results from the fabrication error.

The measured read range at each temperature is about 6 m shorter than the simulated ones, which is due to the imperfect metal contact between the two layers. The loss due to the imperfect metal contact can be modeled by a pair of cascaded resistors between the two layers. Figure 11c shows the read range when the cascaded resistor varies from 0 ohm to 1.2 ohm at 45 °C. When the resistor value is set to 0.8 ohm, the read range at 910 MHz is approximately equal to the measured one. Figure 11d gives the simulated read range when a pair of 0.8 ohm resistors are in series connection with the two metal layers. The measured read range agrees well with the simulated read range.

## 5. Conclusions

In this paper, a high-sensitivity passive sensing antenna with a bimetal strip coil is presented for wireless temperature monitoring. The design utilizes the temperature-dependent deformation characteristic of the bimetal strip to drive the lower feeding part of the dipole rotate, thus changing the resonant frequency. A prototype is designed, fabricated and measured, and the measurement results show that the sensitivity of the proposed antenna is more than 4.00 MHz/°C, which is the highest among the passive temperature-sensing antennas. An approximately linear relationship between the temperature and the resonant frequency is achieved, and the read range is more than 4 m. The proposed sensor is a good candidate for large-scale wireless temperature-sensing networks such as temperature monitoring in greenhouses and livestock farms. Besides, it is noteworthy that the sensing temperature range of the antenna can be enhanced by adopting an appropriate bimetal strip coil and optimizing the dipole antenna parameters.

The imperfect metal contact between the two layers results in a large amount of power loss, which limits the read range and broadens the realized gain bandwidth. If the two parts of the antenna can operate without the requirement of mutual contact, the performance of the sensing antenna would be further improved. 

## Figures and Tables

**Table 1:** Dimensions of the dipole antenna.

Parameter	Value	Parameter	Value
L_0_	12.00 mm	L_9_	3.00 mm
L_1_	6.55 mm	L_10_	3.00 mm
L_2_	3.00 mm	L_11_	4.00 mm
L_3_	9.00 mm	D_0_	2.00 mm
L_4_	3.00 mm	D_1_	20.50 mm
L_5_	3.00 mm	D_2_	3.00 mm
L_6_	2.50 mm	θ_0_	88.1°
L_7_	63.00 mm	θ_1_	135.0°
L_8_	10.00 mm	θ_2_	Variable

**Table 2:** The rotating angle θ_2_ at different temperatures.

Temperature (°C)	θ_2_ (°)
30	0
35	11
40	28
45	45
50	66
